# Physicochemical properties and cell proliferation and adhesive bioactivity of collagen-hyaluronate composite gradient membrane

**DOI:** 10.3389/fbioe.2023.1287359

**Published:** 2023-10-25

**Authors:** Zhaoxuan Li, Xue Song, Yan Fan, Yuming Bao, Hu Hou

**Affiliations:** ^1^ College of Food Science and Engineering, Ocean University of China, Qingdao, Shandong, China; ^2^ Institute of Feed Research of Chinese Academy of Agriculture Sciences, Beijing, China; ^3^ Laboratory for Marine Drugs and Bioproducts, Laoshan Laboratory, Qingdao, Shandong, China

**Keywords:** collagen, guided tissue regeneration, composite gradient membrane, TGF-β/Smad pathway, Wnt/β-catenin pathway

## Abstract

Membrane materials were widely used in guided tissue regeneration (GTR) to prevent fibroblast invasion and form a confined area for preferentially growing of osteoblast. A novel collagen-hyaluronate composite gradient membrane was prepared by Tilapia (*Oreochromis mossambicus*) skin collagen and sodium hyaluronate for potential GTR applications and their bioactivities were investigated by cellular viability. SEM results indicated the membrane showed a dense outer and a porous inner surface for effectively guiding the growth of bone tissue. Physicochemical and biosafety experiments showed the tensile strength of membrane was 466.57 ± 44.31 KPa and contact angle was 74.11°, and the membrane showed perfect biocompatibility and cytocompatibility as well, which met the requirements of GTR material. Cell morphology revealed that the membrane could facilitate the adherence and proliferation of fibroblast and osteoblast. The results of qRT-PCR and ELISA demonstrated that the membrane could effectively activate TGF-β/Smad pathway in fibroblast, and promote the expressions of TGF-β1, FN1 and VEGF. Remarkably, RUNX2 was stimulated in BMP2 pathway by the membrane to regulate osteoblast differentiation. In summary, the collagen-hyaluronate composite gradient membrane not only fulfills the prerequisites for use as a GTR material but also demonstrates substantial potential for practical applications in the field.

## 1 Introduction

Treatment of tissue defects caused by trauma including periodontitis (Mirzaeei, et al., 2022), exodontia (Yan et al., 2022) and tumor resection (Chen, et al., 2022) has always been an important and widely concerned topic in clinical research. Currently, the guided tissue regeneration (GTR) technique has been widely used in treatment of tissue defects (Susanto et al., 2019), based on applying a barrier membrane with multi-function to isolate the wounded-exposed area from soft tissue, which results in the exclusion of epithelial cell migration and prevents the interference of osteoblast proliferation by external fibroblast, achieving osteogenesis (Zhou et al., 2021). According to their degradation properties, GTR membranes are classified as non-absorbable or bio-resorbable membranes. Non-absorbable membranes (e.g., titanium mesh, e-PTEE) have good mechanical properties but require a second surgery to remove, which increases the patients’ pain. Additionally, their poor bioactivity restricts their applications (Yang et al., 2022). On the contrary, bio-resorbable membranes (e.g., polylactic acid, collagen) do not cause secondary surgery and have better biocompatibility than the former, so they have great potential application value in clinic (He et al., 2020a; Song et al., 2022).

Many studies have fabricated and reported that the naturally derived GTR barriers including collagen, hyaluronate, chitosan and their composites could provide space for osteoblast ingrowth and ameliorate the microenvironment for wound healing (Xie et al., 2018; Li et al., 2020). Among, collagen was widely used in GTR due to its good cellular affinity, biocompatibility, non-toxicity and non-immunogenicity (Song et al., 2019). Scaffolds prepared from collagen had the potential to induce rapid migration of fibroblast and support proliferation, osteogenic differentiation and mineralization of hMSCs (Ashworth et al., 2018; de Melo Pereira et al., 2020; Wu et al., 2023). However, collagen-based membranes had performed unsatisfactorily in biodegradability *in vivo* (Bunyaratavej, P., & Wang, H. L., 2001), and thus other bio-polymers were widely added to improve mechanical properties and bioactivities by remodeling their molecular, and hyaluronic acid was a good candidate (Neuman et al., 2015). As a glycosaminoglycan in extracellular matrix (ECM), hyaluronic acid was widely applied in biomedical materials, as a filler in bone cavity, artificial cornea and biomimetic composite material for wound healing recently (Li et al., 2020). Collagen scaffold with hyaluronic acid was prepared by Bavaresco’s group, and the scaffold not only showed better resistance to collagenase and elastic mechanical properties, but also had the ability to support cell attachment and proliferation (Bavaresco, Comín, Salvatierra & Cid, 2020). These results revealed that materials based on collagen and hyaluronic acid could provide the support required in tissue engineering.

Generally, compared with a uniform monolayer membrane, the natural and bionic layered membranes are recommended and mostly studied (Qasim et al., 2015). A dense and microporous surface is needed to prevent fibroblast invading bone-defect area, and a microporous as well as bio-functional interface is required for guiding the regeneration of the missing bone tissue. In the work conducted by Shah et al. (2019), a novel tri-layered, functionally-graded membrane was synthesized through the lyophilization process. Notably, the lower surface of the membrane exhibited a substantial impregnation of osteoblast. However, the mechanism of tissue regeneration guided by membrane materials is unclear.

EDC-NHS crosslinking of fish skin collagen and sodium hyaluronate was used to prepare composite gradient membranes for GTR, and the aim of this study was to explore the microstructure and physicochemical properties of the membrane, and evaluate the biocompatibility and cytocompatibility. And the molecular mechanisms underlying the effects of the COL-HA on fibroblast and osteoblast, such as TGF-β/Smad and Wnt/β-catenin, were explored in-depth.

## 2 Materials and methods

### 2.1 Materials

The frozen skin of Tilapia (*Oreochromis mossambicus*) was obtained from Lushi Aquatic Product Company (Guangzhou, China) and stored at −20°C. Sodium hyaluronate was purchased from Liyang Biological Product Co., Ltd. (Qufu, China). EDC and NHS were purchased from Shanghai Aladdin Biological Technology Co., Ltd. (Shanghai, China). KBr was of spectroscopic grade, purchased from Shanghai Macklin Biochemical Technology Co., Ltd. (Shanghai, China). Actin-Tracker Red-594 and DAPI were purchased from Beyotime Biotechnology Co., Ltd. (Shanghai, China). Other reagents used in this study were of analytical grade, obtained from Sinopharm Chemical Reagent Co., Ltd. (Shanghai, China).

### 2.2 Preparation of collagen from fish skin

Collagen from Tilapia skin was prepared according to the method of Song et al. (2022), with a slight modification. Briefly, the skin was treated with 0.1M NaOH (1:30, w/v) for 24 h and then immersed in 0.5 M acetic acid (1:40, w/v), containing 1% (w/w) pepsin for 48 h with stirring, and centrifuged at 9,000 ×g (2-16KL, Sigma, Germany) for 30 min. The supernatant was collected and salted out by adding NaCl at a final concentration of 1.0 M. The precipitate was dissolved in 0.5 M acetic acid by centrifugation at 10,000 ×g for 30 min, dialyzed for 5 d and freeze-dried to obtain collagen.

### 2.3 Preparation of collagen membrane (COL)

Collagen membrane was prepared according to the method of Sun et al. (2020), with a slight modification. Collagen solution (10 mg/mL) was poured into 24-well plates and freeze-dried for 24 h. The cross-linking solution of 60 mM EDC and 30 mM NHS in 50 mM MES was prepared and the membrane was immersed in the cross-linking solution for 4 h. After freeze-drying, the crosslinked collagen membrane was obtained and named as COL.

### 2.4 Preparation of collagen-hyaluronate composite gradient membrane (COL-HA)

**FIGURE 1:**
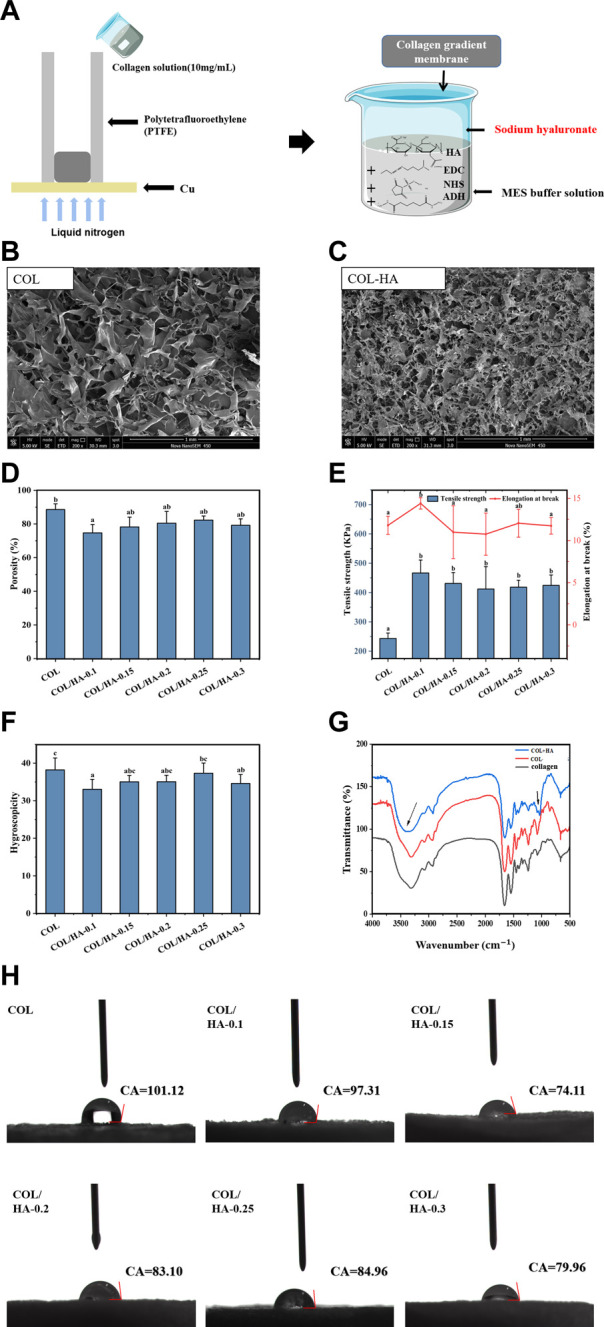
Schematic illustration for the preparation of COL-HA, its SEM images and physicochemical properties of COL and COL-HA. **(A)** Schematic illustration for the preparation of COL-HA; **(B)** SEM image of COL; **(C)** SEM image of COL-HA; **(D)** Porosity of COL and COL-HA; **(E)** Tensile strength and elongation at break of COL and COL-HA; **(F)** Swelling property of COL and COL-HA; **(G)** FTIR spectra of collagen, COL and COL-HA; **(H)** contact angles of COL and COL-HA. Data shown represent mean ± SE in each group. Different small letters represent significant differences between groups (*p* < 0.05).

As shown in Figure 1A, collagen-hyaluronate composite gradient membrane (COL-HA) was prepared through unidirectional freeze-drying and cross-linking technology. Firstly, the collagen solution (10 mg/mL) was poured into the mold which was surrounded by PTFE pipe and the bottom was copper plate and the bottom of the mold was in direct contact with liquid nitrogen to freeze the collagen solution quickly. After freeze-drying, the gradient collagen membrane was obtained. Then, HA crosslinking solution was prepared with 60 mM EDC, 30 mM NHS, sodium hyaluronate and excess adipyl hydrazide. The membrane was immersed in the crosslinking solution for 4 h, and freeze-dried for 24 h. It was treated with HA crosslinking solutions to obtain collagen-hyaluronate composite gradient membranes, until the final ratios of collagen to hyaluronate in membranes were 1:0.1, 1:0.15, 1:0.2, 1:0.25 and 1:0.3, respectively, named as COL/HA-0.1, COL/HA-0.15, COL/HA-0.2, COL/HA-0.25 and COL/HA-0.3.

### 2.5 SEM of COL-HA

The morphology of the membrane was observed at ×200 magnification by SEM (JSM-840, JEOL Ltd., Tokyo, Japan) with an accelerating voltage of 5.0 kV after the sputter-coated with gold (He et al., 2020b).

### 2.6 Porosity measurement of COL-HA

The porosity of the membrane was tested by the ethanol replacement method (Chen et al., 2020). The membrane was immersed in a volume of anhydrous ethanol and vacuum-repressure cycled 3 times by a vacuum drying oven (DZX-1, Shanghai Fuma Laboratory Instrument Co., Ltd., China) to facilitate the penetration of ethanol into the pores of the membrane without causing shrinkage or swelling materials.

Porosity was calculated according to the following equation.

$$Porosity\left(\%\right)=\frac{V_{1}-V_{3}}{V_{2}-V_{3}}\times 100$$ (1)

where “*V*_*1*_” was initial volume of anhydrous ethanol (mL); “*V*_*2*_” was the volume of the ethanol containing the sample (mL); “*V*_*3*_” was Volume of anhydrous ethanol after sample removal (mL).

### 2.7 Mechanical, swelling and hydrophilic properties of COL-HA

#### 2.7.1 Mechanical properties of COL-HA

Mechanical properties of the membrane were evaluated by texture analyzer (TMS-Pro, Food Technology Corporation, USA) (Sun et al., 2018). The membrane was cut into appropriate size, clamped to sample holder, and tested with the speed of 60 mm/min and displacement of 10 mm. Each measurement was performed in sextuplicate. Results were reported as the mean standard deviation. Tensile strength (Ts) was calculated as follows:

$$Ts=\frac{F}{S}\times 1000$$ (2)

where “*F*” was the maximum tension at the instant of fracture of the sample N); “*S*” was the cross-sectional area of the sample (mm^2^).

Elongation at break (EAB) was calculated as follows:

$$EAB\left(\%\right)=\frac{∆L}{L}\times 100$$ (3)

where “*ΔL*” was the displacement of extension when the sample was broken (mm); “*L*” was the gauge distance (mm).

#### 2.7.2 Swelling property of COL-HA

The Swelling property of the membrane was tested by the method of Sun et al. (2017) with minor modification. The membrane was weighed at first, and then immersed in solution containing 142 mM Na^+^ and 2.5 mM Ca^2+^ for 5 h at 37°C. The wet membrane was placed in the filter screen to remove the surface water, and weighed immediately. Water absorptivity ratio of the membrane (WAR) was determined from the following equation:

$$WAR=\frac{W_{1}-W_{0}}{W_{0}}$$ (4)

where “*W*_*0*_” was the dry weight of membrane g) and “*W*_*1*_” was the wet weight of membrane (g).

#### 2.7.3 Surface contact angle characterization of COL-HA

Hydrophilicity of the membrane was examined on a contact angle measurement instrument (JC2000DM, Shanghai, China). Deionized water was dropped onto each group of membranes (*n* = 5), and the average readings were recorded. The contact angle was calculated using the Auto FAST algorithm, a common and efficient algorithm for image autofocusing within the image analysis software.

### 2.8 Fourier transform infrared (FT-IR) analysis of COL-HA

The samples and spectral grade KBr were mixed and ground at a ratio of 1:50 before examining the functional groups and their changes through FT-IR spectroscopy (iS10, Thermo-Nicolet Co. Ltd., USA). The FT-IR spectra was acquired in the range of 4,000–500 cm^-1^and the data was analyzed by OMNIC 9.0 software according to the method of Sun et al. (2017).

### 2.9 Evaluation of cells proliferation and adhesion on COL-HA

#### 2.9.1 Cell culture

Mouse fibroblast and osteoblast were cultured into 7.5 mL culture flasks in DMEM with 10% fetal bovine serum (FBS) and 1% penicillin/streptomycin (P/S) in an incubator filled with 5% CO_2_ at 37°C, respectively. Upon reaching confluence, cells were trypsinized from the culture flask and counted with a hemocytometer. Subsequently, cell suspensions of fibroblasts and osteoblasts were prepared and seeded onto appropriate membranes and well plates. All the experiments were performed using cells at passages 4-8.

#### 2.9.2 Phalloidin staining protocol

The membranes were sterilized by ^60^Co for 72 h, and preconditioned in DMEM containing 10% FBS and 1% P/S for 48 h. The fibroblast (1×10^4^/mL) and osteoblast (1×10^4^/mL) were seeded onto each membrane and incubated in an incubator filled with 5% CO_2_ at 37°C for 72 h. The control group (without membranes) was seeded with equal numbers of cells and cultured under the same conditions.

After 72 h, the culture media was removed. The fibroblast and osteoblast were fixed with 4% paraformaldehyde for 15 min at 25°C and then permeabilized 3 times with 0.5% Triton-X for 5 min each. Cells were stained with Actin-Tracker Red-594 at 37°C for 40 min, and then stained with DAPI for 2 min at 25°C. Cells were imaged using a fluorescent microscope (Ni-E, NIKON, Japan) (Li et al., 2012).

### 2.10 Acute systemic toxicity

Acute systemic toxicity of the membrane was performed according to the GB/T 16886.11-2011. Twenty male KM mice were randomly divided into four groups. COL extract, COL-HA extract, saline (negative control) and 10% phenol aqueous solution (positive control, 0.45%, w/v) were injected intraperitoneally at dose of 20 mg/kg body weight, respectively. The sample-related toxic symptoms such as respiration, physical activity, body weight and mortality rate of the mouse were observed at 24, 48 and 72 h after injection. All animal experiments were carried out in accordance with the guidelines of the Scientific Ethics Special Committee of the Academic Committee of the Ocean University of China.

### 2.11 Intradermal stimulation test

Intradermal stimulation test of the membrane was performed according to the GB/T 16886.10-2017. Five male SD rats were injected intraperitoneally with 10% chloral hydrate (3 mL/kg). The hair on the back was shaved to expose the experimental area and the skin was disinfected with 75% ethanol. Four spots were taken at the same intervals on the back and injected with 100 μL of COL extract, COL-HA extract, saline (negative control) and 10% methanol solution (positive control), respectively. Erythema, eschar and edema were recorded at 24, 48 and 72 h after injection.

### 2.12 Hemolysis ratio

Hemolysis test was performed according to method of He et al. (2020a). To hemocyte suspension, fresh anticoagulated rat blood should be diluted with saline in a 5:4 ratio. COL and COL-HA were cut into 10 mm × 10 mm size, immersed in 10 ml of saline for 30 min at 37°C. Then, a quantity of 200 μL of hemocyte suspension was added to 10 mL saline with membranes (COL and COL-HA) and incubated for 1 h at 37°C. An equal volume of hemocyte suspension was added into distilled water and saline as positive and negative control groups, respectively. Finally, samples were pre-centrifuged at 2,000 ×g for 5 min, and the absorbance of supernatant read at 540 nm. The hemolysis ratio (HR) was determined from the following equation:

$$Hemolysis\;ratio\;\left(\%\right)=\frac{D_{s}-D_{nc}}{D_{pc}-D_{nc}}\times 100$$ (5)

where *D*_*s*_, *D*_*nc*_, and *D*_*pc*_ are the absorbance values of the sample, negative control, and positive control, respectively.

### 2.13 qRT-PCR

The fibroblast (1×10^5^/mL) and osteoblast (1×10^5^/mL) were seeded onto each membrane and incubated in an incubator filled with 5% CO_2_ at 37°C for 7 days. Total RNA was extracted from fibroblast and osteoblast using Trizol reagent (Vazyme, China) and then reverse transcribed into cDNA (Wu et al., 2020). After that, cDNA was used for qRT-PCR in a LineGene 9,600 Plus system with the SYBR^®^ Green Realtime PCR Master Mix. Amplifications were performed in a program for 40 cycles as follows: first cycle (30 s at 95°C, 5 s at 95°C, 10 s at 55°C, 15 s at 72°C) and the next 39 cycles (5 s at 95°C, 10 s at 55°C, 15 s at 72°C). The gene-specific primers were listed in Table 1.

**TABLE 1:** Primer Sequences of qRT-PCR.

Gene	Forward	Reverse
TGF-β1	ACC​GCA​ACA​ACG​CCA​TCT​ATG​AG	GGC​ACT​GCT​TCC​CGA​ATG​TCT​G
TGF-βr1	CAT​TGC​TGG​TCC​AGT​CTG​CTT​CG	TGG​TGA​ATG​ACA​GTG​CGG​TTA​TGG
SMAD2	ATG​TCG​TCC​ATC​TTG​CCA​TTC​ACT​C	ATG​TCG​TCC​ATC​TTG​CCA​TTC​ACT​C
SMAD3	AGA​CGC​CAG​TTC​TAC​CTC​CAG​TG	GCC​AGC​AGG​GAA​GTT​AGT​GTT​CTC
SMAD4	TGT​TGT​GAC​TGT​GGA​TGG​CTA​TGT​G	CTC​GCT​CTC​TCA​ATC​GCT​TCT​GTC
COL1a1 (fibroblast)	GAC​AGG​CGA​ACA​AGG​TGA​CAG​AG	CAG​GAG​AAC​CAG​GAG​AAC​CAG​GAG
FN1	CCG​TGG​ACC​AGG​TTG​ATG​ATA​CTT​C	GCT​CTG​TGC​TAC​TGC​CTT​CTA​CTG
VEGF	GGG​CTC​TTC​TCG​CTC​CGT​AGT​AG	CCC​TCT​CCT​CTT​CCT​TCT​CTT​CCT​C
bFGF	GAG​CGA​CCC​ACA​CGT​CAA​ACT​AC	CAG​CCG​TCC​ATC​TTC​CTT​CAT​AGC
LRP5	CAA​GAC​GAT​CAG​CCG​AGC​CTT​C	CAT​CCA​GTC​CAC​AGC​CAT​TCC​TTC
GSK3β	GGC​TGT​GTG​TTG​GCT​GAA​TTG​TTG	TTT​GCT​CCC​TTG​TTG​GTG​TTC​CTA​G
β-catenin	GCT​GCT​GTC​CTA​TTC​CGA​ATG​TCT​G	GGC​ACC​AAT​GTC​CAG​TCC​AAG​ATC
TCF-1	CAA​GAC​GAT​CAG​CCG​AGC​CTT​C	CAT​CCA​GTC​CAC​AGC​CAT​TCC​TTC
RUNX2	GAT​GAT​GAC​ACT​GCC​ACC​TCT​GAC	TGA​GGG​ATG​AAA​TGC​TTG​GGA​ACT​G
BMP2	AGA​TCT​GTA​CCG​CAG​GCA​CT	GTT​CCT​CCA​CGG​CTT​CTT​CG
ALP	CGG​CGT​CCA​TGA​GCA​GAA​CTA​C	CAG​GCA​CAG​TGG​TCA​AGG​TTG​G
COL1a1 (osteoblast)	CAA​GAC​GAT​CAG​CCG​AGC​CTT​C	CAT​CCA​GTC​CAC​AGC​CAT​TCC​TTC
OPN	ATG​GAC​GAC​GAT​GAT​GAC​GAT​GAT​G	CTT​GTG​TAC​TAG​CAG​TGA​CGG​TCT​C

### 2.14 Measurement of related cytokine concentrations

The concentrations of TGF-β, FN, VEGF, BMP2, β-catenin and ALP by fibroblast and osteoblast in the culture medium were measured, using commercially available ELISA kits (Cusabio, Wuhan, China) according to the manufacturer’s instructions.

### 2.15 Statistical analysis

All experiments were conducted with at least three replicates. The results were shown as mean ± SD and a level of *p* < 0.05 was considered significant.

## 3 Results and discussion

### 3.1 Morphological analysis and characterization of COL-HA

Porosity and pore size of biomaterial materials played a critical role in tissue and bone formation *in vitro* and *in vivo* (Karageorgiou and Kaplan, 2005). Collagen, as the significant component of the extracellular matrix (ECM), might be the ideal material. It had a native surface to favor cellular attachment as well as being chemotactic to cells (Sachlos et al., 2003). The effect of HA cross-linking of collagen on the microstructure of the membrane by scanning electron microscope (SEM) was shown in Figures 1B, C. The results clearly showed that the pore structure became more distorted when collagen was cross-linked with HA, as proven by extensive side-branches within the primary pores.

Similarly, Li et al. (2020) reported that the side-branches were observed in collagen-hyaluronic acid scaffolds and the growth of secondary pores had led to a reduction in the average pore size of collagen-hyaluronic acid scaffolds. Therefore, the addition of HA would affect the density and pore size of collagen matrix. As a consequence of alterations in the internal molecular structure of the membranes, there arises a necessity for comprehensive assessments encompassing their porosity, mechanical characteristics, and cellular activity, which is imperative to determine the feasibility of employing composite gradient membranes within the domain of guided tissue regeneration.

### 3.2 Characterization of porosity of COL-HA

The porosity arose from the formation of ice crystals during the freezing of collagen solution, forcing the collagen to aggregate into interstitial spaces and creating a network of interconnected fibrils (Kleinman et al., 1981; Park et al., 2014). The pores were connected to each other, allowing infiltration of cell culture media nutrients and intermingling between cell colonies (Luo et al., 2014). The influence of different proportions of HA on porosity of collagenous materials was investigated in detail. Figure 1D showed the porosity of COL-HA with different proportions, and the addition of HA decreased the porosity of COL-HA. COL showed the highest porosity (88.57% ± 3.39%), while COL/HA-0.1 showed the lowest porosity (74.72% ± 5.00%), indicating that the addition of HA led to a reduction in porosity, which was consistent with experimental results of Li et al. (2020).

Park et al. (2002) produced porous collagen/hyaluronic acid scaffolds cross-linking with EDC by freeze-drying at −196°C, −70°C and −20°C resulting in 62%, 62% and 64% porosities, respectively, which also retained the porous structure. The formation of a large number of fibrous side branches in COL-HA might be an essential reason for the decrease of porosity.

### 3.3 Changes of mechanical properties, swelling, and hydrophilic properties of the membranes

As biomaterials used in tissue engineering, tensile strength and elongation at break were the main mechanical indexes (Susanto et al., 2019). Figure 1E illustrated the mechanical strength changes of membranes. The addition of HA significantly affected the mechanical properties of the collagen membrane, and the tensile strength increased 1.7-1.9 times. The maximum tensile strength of COL/HA-0.1 group was 466.57 ± 44.31 KPa, while the elongation at break was 14.40% ± 0.67%. The reason was that EDC could cross-link COL between carboxylic and amine groups through the formation of amide (Gurumurthy and Janorkar, 2021), and our data were in agreement with previous observation.

As a biomedical material, the swelling property of the membrane is an important index in assessing the efficacy of the material. As shown in Figure 1F the pure collagen membrane itself had a high liquid absorbability, and water absorptivity ratio of COL was 38.20 ± 2.16 multiple, but HA cross-linking resulted in a reduction in the liquid absorption of COL-HA, which may be related to a decrease in the porosity of the membrane. The liquid absorbability of materials decreased with the increase of the degree of crosslinking, due to the formation of ester and amide bonds between collagen and HA through cross-linking, resulting in a significant reduction in the content of free carboxyl groups (Kaiser et al., 2017).

Biomaterials with favorable hydrophilic surfaces can facilitate cell adhesion and growth (Bian et al., 2020). As shown in Figure 1H, when collagen was cross-linked with HA and EDC, the contact angle of COL-HA decreased, and the minimum contact angles of COL/HA-0.15 group was 74.11°, indicating that the wettability of COL-HA was enhanced by compounding HA. The collagen-glycosaminoglycan membrane prepared by Ruozi et al. (2009) was more hydrophilic than the pure collagen membrane, creating a good microenvironment that favored the proliferation of fibroblast and keratin-forming cells. Owing to the hydrophilicity of HA, cross-linking of collagen with HA led to significant reduction in the contact angle, which was more conducive to cell adhesion and proliferation.

### 3.4 FT-IR analysis of COL-HA

The influence of HA and EDC-NHS cross-linking on the secondary structure and functional groups of collagen membranes was analyzed by Fourier transform infrared spectroscopy. Figure 1G showed FT-IR spectra of the collagen, COL and COL-HA. Compared to the collagen, COL showed the characteristic peaks of amide A (3,316 cm^-1^), amide B (2,932 cm^-1^), amide I (1,653 cm^-1^), amide II (1,540 cm^-1^), and amide III (1,244 cm^-1^), similar to the study of Nair et al. (2020).

The amide A band of COL-HA (3,315 cm^-1^) showed a broader peak shape and generated bathochromic shift, which was caused by the polysaccharide’s C-O stretching vibration and the N-H stretching vibration of the N-acetyl side chain (Mader et al., 2016). In addition, carboxyl group of hyaluronic acid could be activated by EDC before reacting efficiently with the amino compound to form an amide bond, further explaining the enhanced mechanical strength (Park et al., 2002).

### 3.5 Cell morphology of fibroblast and osteoblast cultured on COL-HA

**FIGURE 2:**
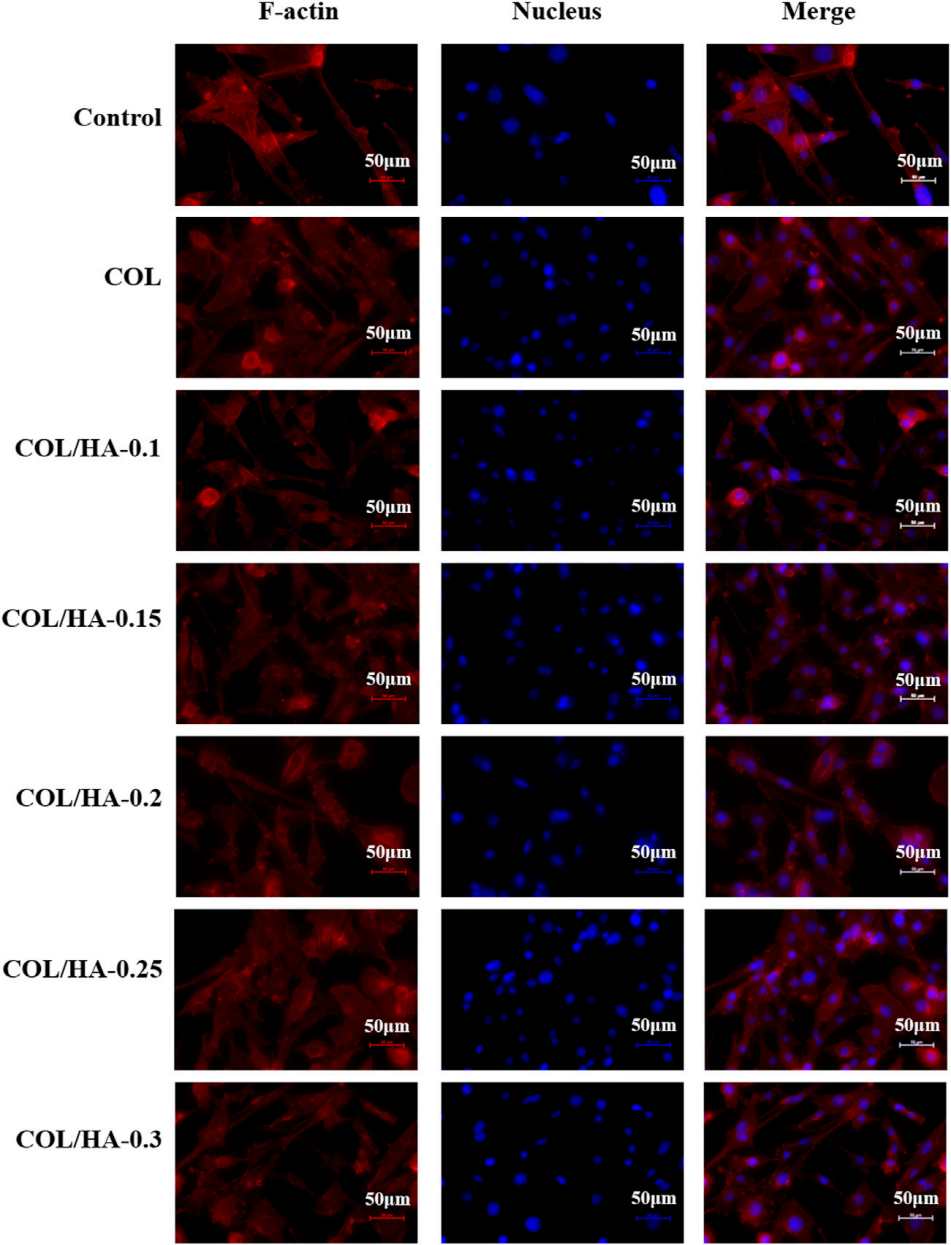
The adhesion of fibroblast cultured on COL and COL-HA at 72 h. The nucleus was stained blue and F-actin was stained red. Fibroblast seeded into COL-HA was mostly spindle-shaped, polygonal and flattened star-shaped, with clearly visible pseudopods and well-spreading.

**FIGURE 3:**
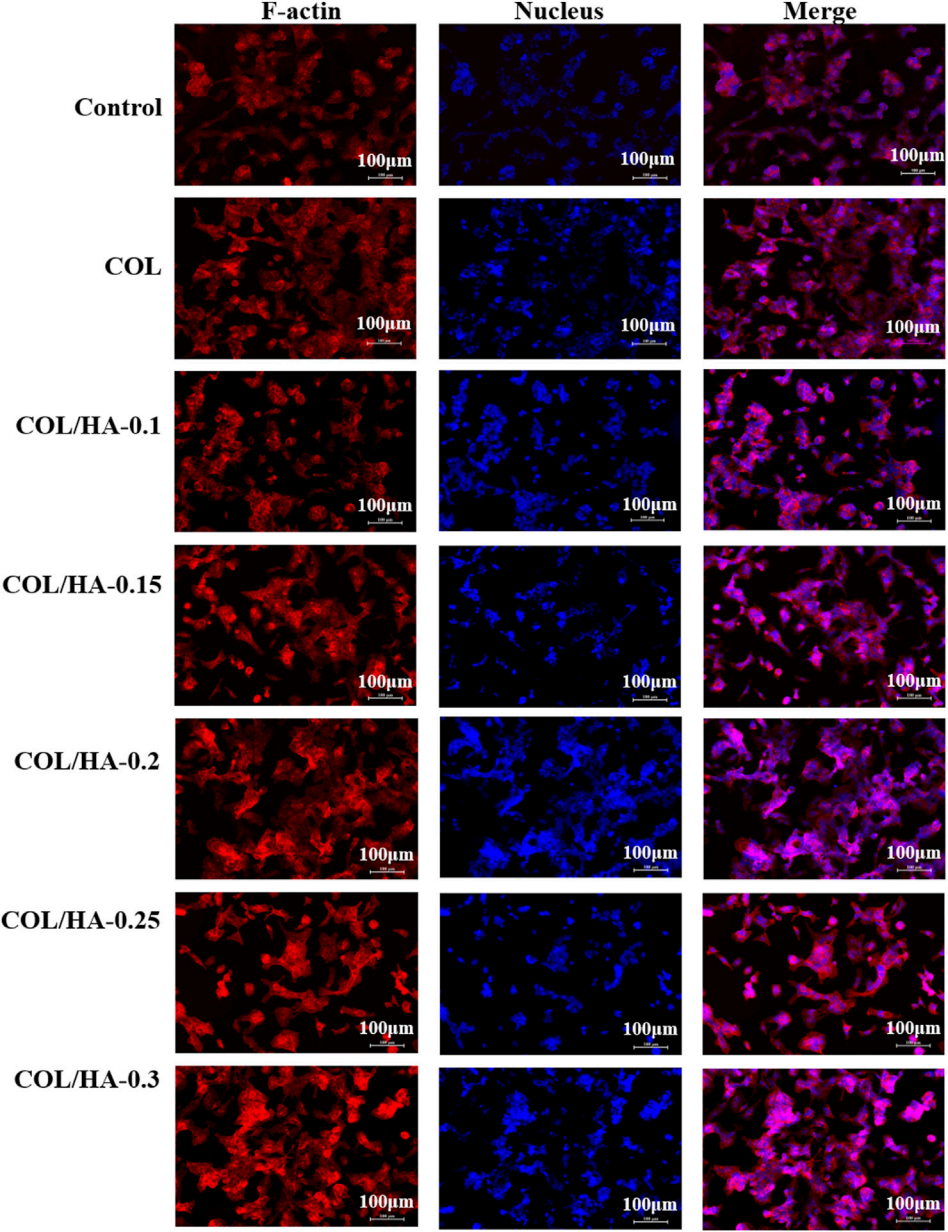
The adhesion of osteoblast cultured on COL and COL-HA at 72 h. Osteoblast seeded into COL-HA presented spindle or cone-shaped distribution with high intercellular density and favorable cell growth.

Actin-Tracker Red-594/DAPI staining was employed to visualize and evaluate fibroblast and osteoblast adhesion on COL-HA. Figure 2 and Figure 3 showed the cell morphology of fibroblast and osteoblast cultured on different proportions of COL-HA membranes for 72 h.

As shown in Figure 2, fibroblast was mostly spindle-shaped, polygonal and flattened star-shaped, with clearly visible pseudopods and well-spreading. Collagen could provide a better microenvironment for cellular activities. Novel fibrous collagen-based cream prepared by Udhayakumar et al. (2017) enhanced the cell-collagen and cell-cell interactions and thus was compatible with the growth and proliferation of fibroblast. Shepard et al. (1996) proposed that the physiological effects of HA might result from its ability to function as a co-receptor in the ligand-binding pathway, not only combining growth factors to increase the effective concentration of these factors on the cell surface, but also attracting integrins to the cell surface to promote cell proliferation.

As shown in Figure 3, osteoblast presented spindle or cone-shaped distribution with high intercellular density and favorable cell growth. Shi et al. (2013) prepared collagen membranes as pseudo-periosteum loaded stem cells and endothelial cells, and observed notable osteogenesis compared with pseudo-periosteum-free scaffolds. COL-HA had better cytocompatibility in cell morphology and proliferation than COL due to better hydrophilicity. Li et al. (2012) found that the COL/HA PEM coating could accelerate the proliferation and differentiation of preosteoblast, because of the good hydrophilic properties of collagen and HA. Therefore, biological evaluation *in vitro* demonstrated that COL-HA could promote the growth and adhesion of fibroblast and osteoblast.

### 3.6 Biocompatibility evaluation of COL-HA

**FIGURE 4:**
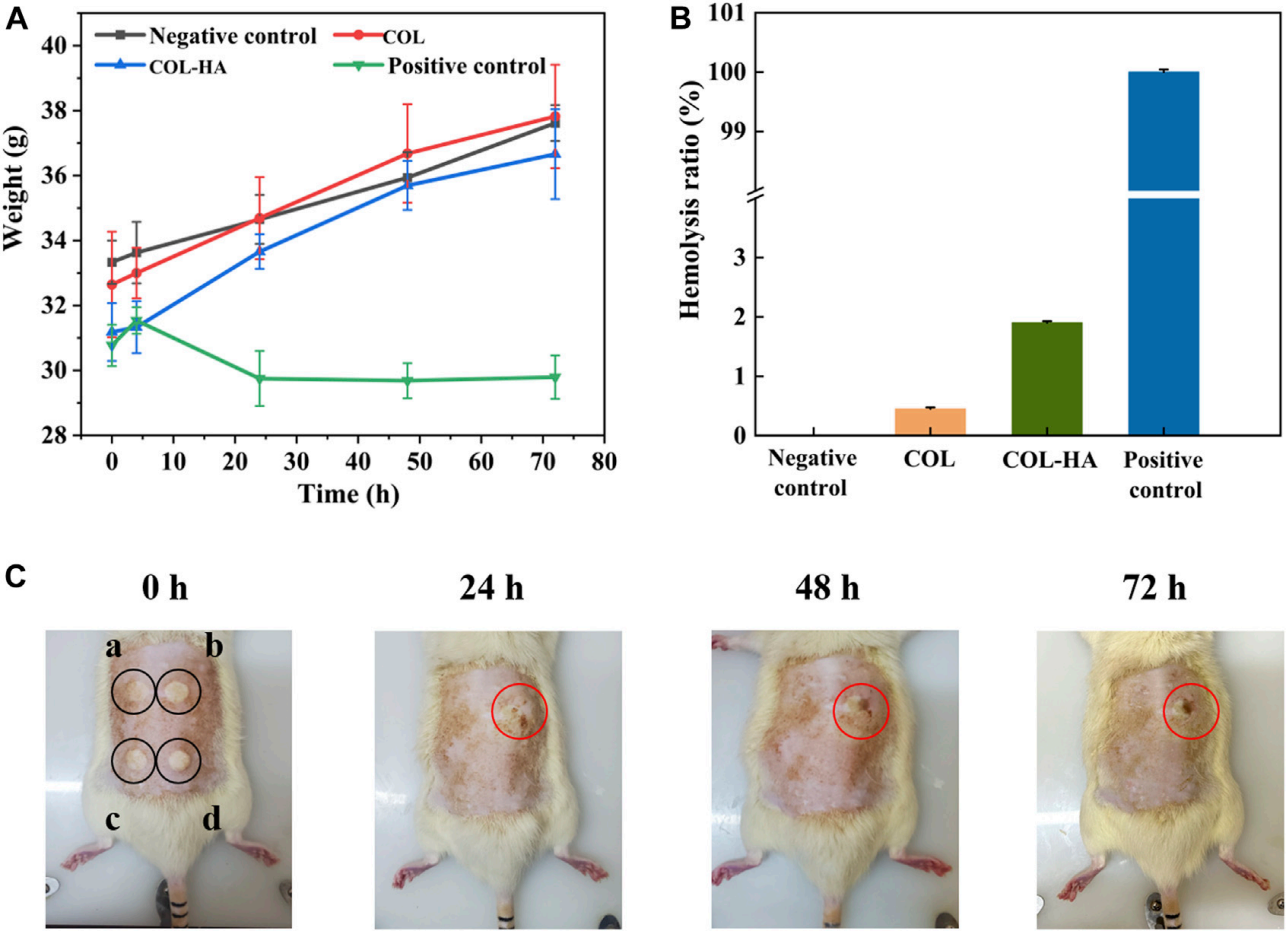
Biocompatibility evaluation of COL and COL-HA. The test results indicated that COL-HA exhibited biocompatibility and showed no significant biotoxicity. **(A)** Changes of body weight in acute systemic toxicity assay; **(B)** hemolysis ratio of COL and COL-HA; **(C)** dermal irritation test (point “a, b, c, d” were injected normal saline, formaldehyde solution, COL extract and COL-HA extract, respectively).

The acute systemic toxicity test was used to evaluate the adverse effects that develop after single, multiple or continuous exposure to the test sample within 24 h, which was an important index of bio-safety evaluation for biomedical materials (Song et al., 2022). As shown in Figure 4A, the body weights for the experimental and control groups were summarized. No adverse effects were observed between the experimental groups and the negative control group, and the weight of the experimental groups and the negative control group increased over time. However, for phenol group, the symptoms of intoxication and significant body weight losses were observed. Therefore, our inference suggested that COL-HA administration did not induce acute systemic toxicity in the murine model.

Hemolysis experiment was one of the most common methods to study the compatibility of biomaterials with blood cells (Ma et al., 2019). Figure 4B showed the hemolysis rate of the samples. The hemolysis ratio of COL and COL-HA were 0.65% ± 0.30% and 1.06% ± 0.17%, respectively, lower than the safe value of 5%, and meant that COL-HA had no obvious destructive effect on erythrocyte, indicating that COL-HA did not lead to severe hemolysis.

Intradermal reaction test was performed to assess the potentiality of irritation reaction of the biomedical materials through the injection of leach liquor (Park et al., 2019). As shown in Figure 4C, COL, COL-HA and the negative control groups did not show any erythema and eschar or edema on the skin. In contrast, erythema and eschar were observed on the skin in the positive control group and did not subside within 72 h. The results denoted that COL-HA extract had no obvious skin irritation, which was consistent with the fact that COL-HA had low antigenicity.

### 3.7 TGF-β/Smad signaling pathway in fibroblast

**FIGURE 5:**
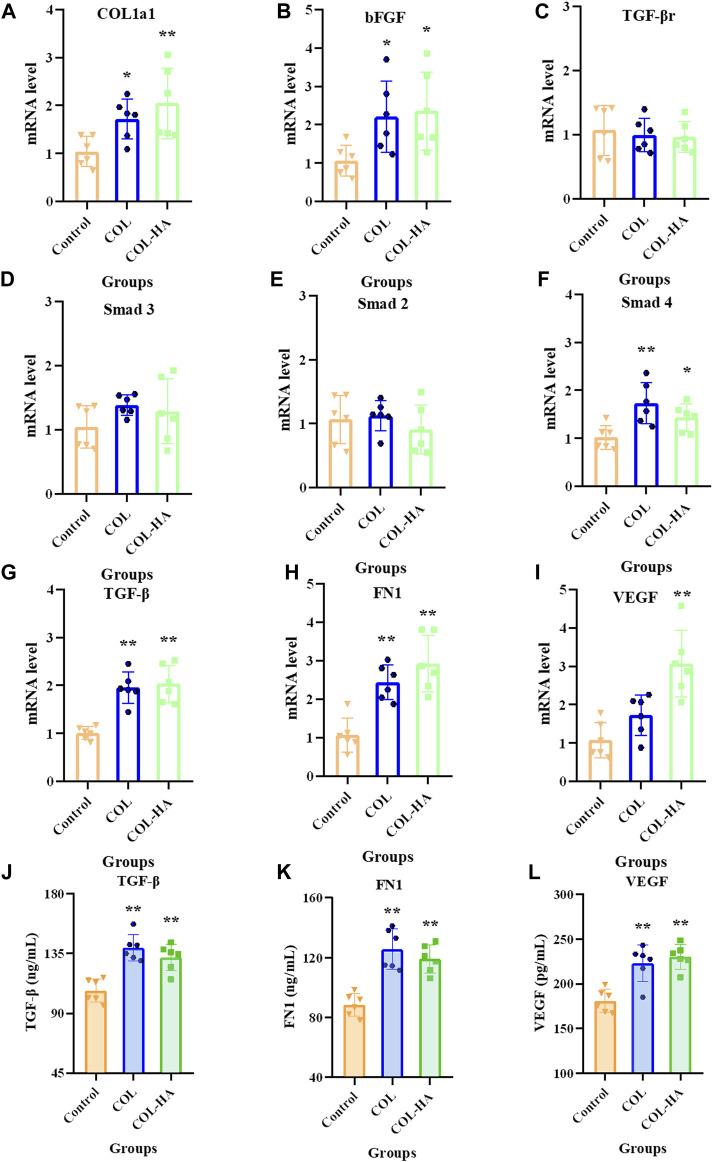
mRNA levels of COL1a1**(A)**, bFGF **(B)**, TGF-βr **(C)**, Smad3 **(D)**, Smad2 **(E)**, Smad4 **(F)**, TGF-β **(G)**, FN1 **(H)** and VEGF **(I)** relative to β-actin in fibroblast cultured on the surfaces of COL, COL-HA and the untreated coverslips (the control group) at 7 d. The protein concentrations of TGF-β **(J)**, FN1 **(K)** and VEGF **(L)** in culture medium was determined when fibroblast was cultured on the surfaces of COL, COL-HA and the untreated coverslips (the control group) at 7 d. *p < 0.05 vs control. **p < 0.01 vs control.

TGF-β is an essential inducer of fibroblast differentiation affecting cell types that are involved in all stages of wound healing and works through the Smad signaling pathway. TGF-β/Smad signaling pathway is related to the Type I collagen formation during wound healing (Schiller et al., 2004; Li et al., 2016; Xie et al., 2018). As shown in Figure 5, compared to the control group, COL-HA significantly promotes the mRNA level of COL1a1 and bFGF by 96.55% and 121.89% (*p* < 0.05). bFGF stimulates the proliferation and differentiation of cells and promotes the growth of granulation tissue, thereby accelerating the wound healing (Xu et al., 2019). As shown in Figures 5J–L, compared with the control group, the expressions of TGF-β, FN1 and VEGF in COL-HA group were increased by 23.15%, 34.62% and 27.20%, respectively, consistent with the increasing trend of mRNA levels of these factors, which could effectively accelerate the wound healing. Similarly, THP-1 cells seeded on EDC crosslink collagen scaffolds secreted significant amounts VEGF, which regulated a variety of pathways in wound healing, including revascularisation, as well as collagen synthesis (Li et al., 2012).

The mRNA levels of TGF-β and Smad4 were significantly increased in the COL-HA group by 121.74% and 39.84% (*p* < 0.05), while TGF-βr, Smad2 and Smad3 showed no significant changes (*p* > 0.05). Therefore, it can be speculated that COL-HA could activate the TGF-β/Smad signaling pathway in fibroblast to promote the secretion of COL1a1, FN1, VEGF and bFGF during tissue regeneration. The regulation of COL-HA on TGF-β/Smad pathway of fibroblast was shown in Figure 7A.

### 3.8 Wnt/β-catenin signaling pathway in osteoblast

**FIGURE 6:**
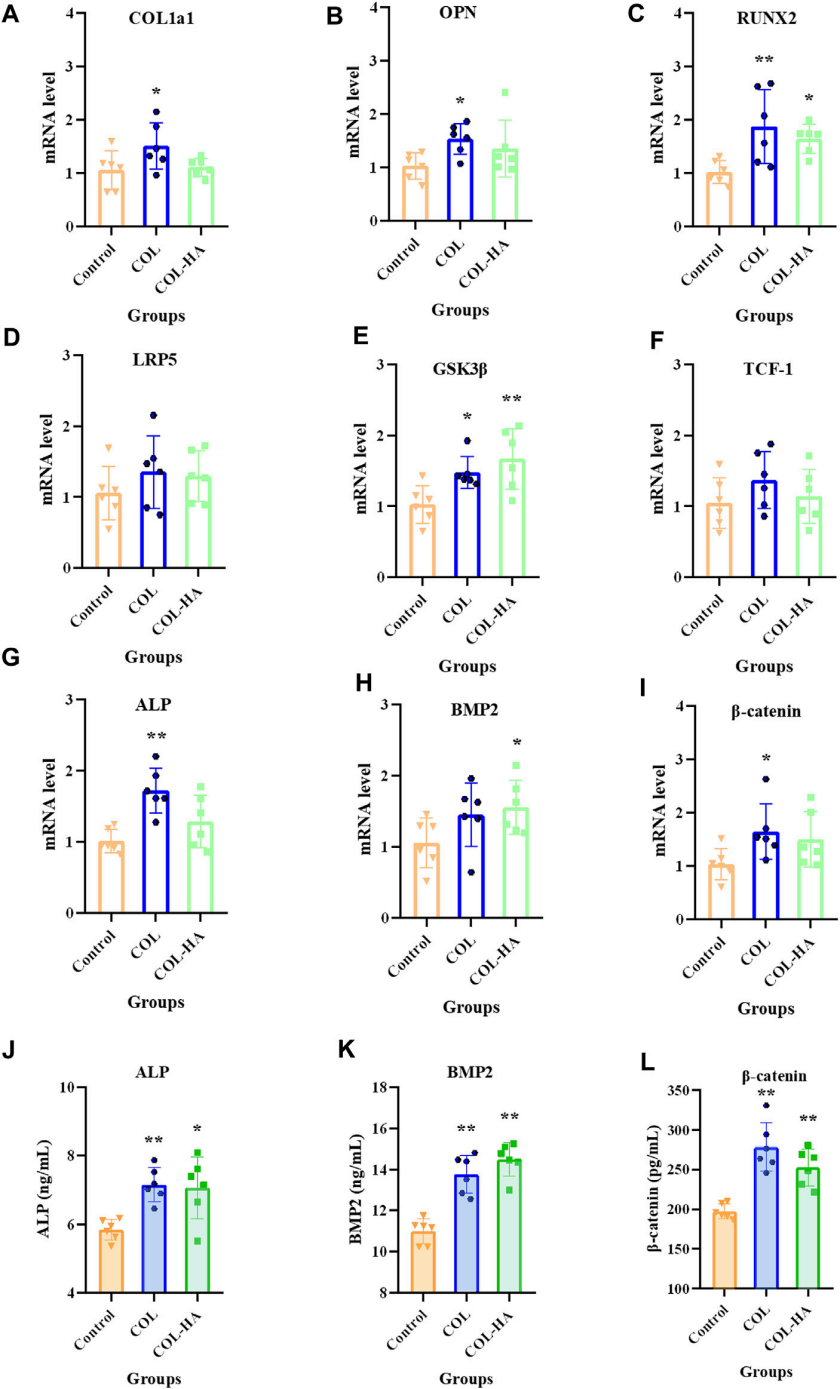
mRNA level of COL1a1 **(A)**, OPN **(B)**, RUNX2 **(C)**, LRP5 **(D)**, GSK-3β **(E)**, TCF-1 **(F)**, ALP **(G)**, BMP2 **(H)** and β-catenin **(I)** relative to β-actin in osteoblast cultured on the surfaces of COL, COL-HA and the untreated coverslips (the control group) at 7 d. The protein concentrations of ALP **(J)**, BMP2 **(K)** and β-catenin **(L)** in culture medium was determined when osteoblast was cultured on the surfaces of COL, COL-HA and the untreated coverslips (the control group) at 7 d. *p < 0.05 vs control. **p < 0.01 vs control.

During early osteogenesis, osteoblast produce extracellular matrix proteins, including COL1a1, RUNX2, BMP2, and ALP, which induce osteoblast deposition and mineralization (Yoon et al., 2013). The Wnt/β-catenin signaling pathway positively regulates the quality of bone tissue formation (Lin et al., 2016). As shown in Figure 6, compared to the control group, the expressions of ALP and BMP2 were significantly upregulated by 21.15% and 16.47% in osteoblast in COL-HA thereby guiding bone regeneration (*p* < 0.05), consistent with the increasing trend of mRNA levels. RUNX2 mRNA was notably upregulated by 60.94% in COL-HA group (*p* < 0.05). The expression of β-catenin in COL-HA was upregulated by 28.12% (*p* < 0.01). However, there were no significant differences in the mRNA levels of LRP5 and TCF-1 between COL-HA and control group (*p* > 0.05). In the study conducted by Toosi et al. (2022), the observation of the effective augmentation of osteogenic lineage differentiation in mesenchymal stem cells by collagen scaffold nanoparticles was made. In the research performed by Zhang et al. (2018), the proposal that a substantial capacity for promoting bone regeneration through the activation of the Wnt signaling pathways was demonstrated by collagen-based scaffolds. Additionally, the documentation of the role of BMP2 in the promotion of RUNX2 expression was carried out by Phimphilai et al. (2006).

**FIGURE 7:**
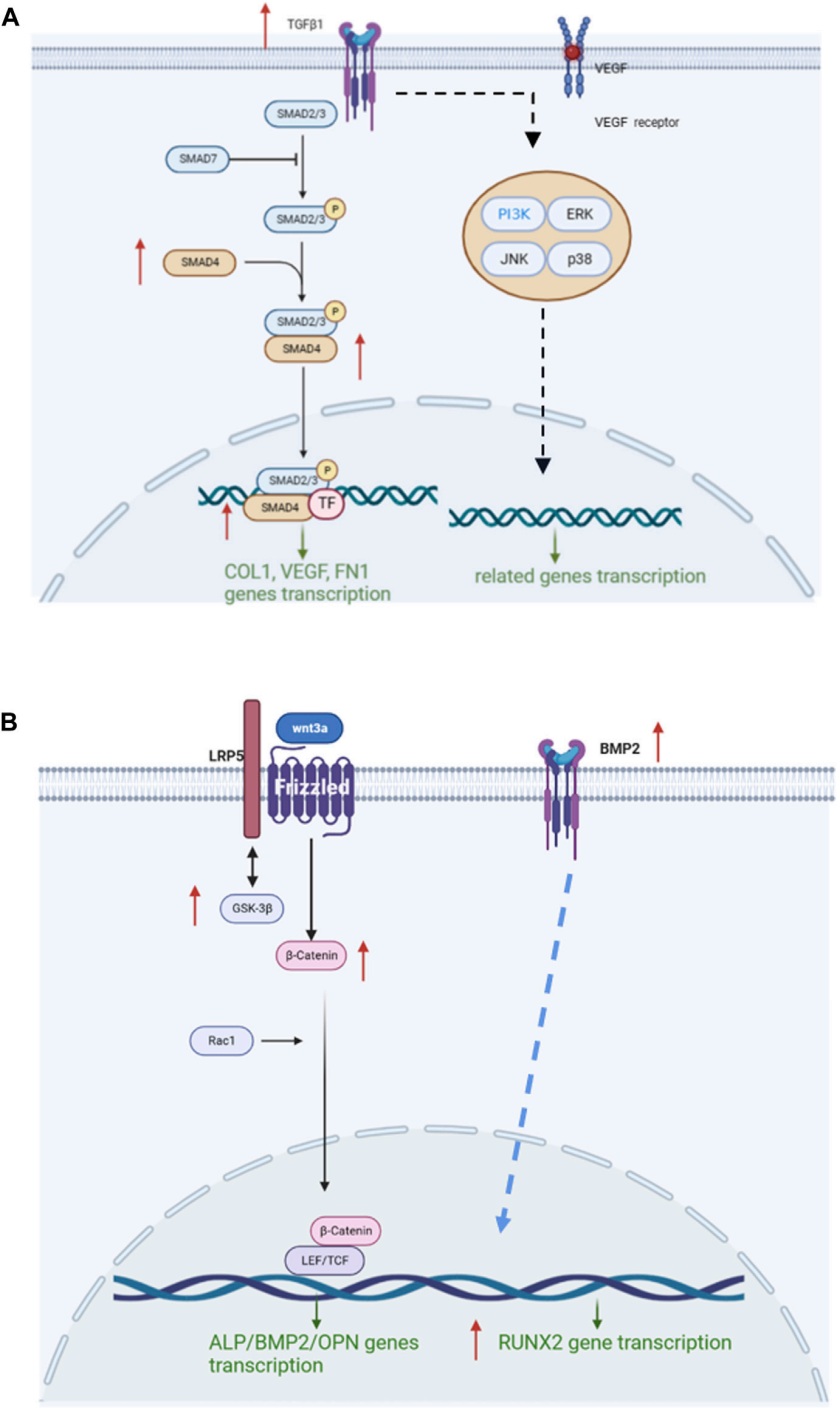
Mechanism of COL-HA on fibroblast and osteoblast. **(A)** The regulation of COL-HA on TGF-β/Smad pathway of fibroblast. **(B)** The effect of COL-HA on Wnt/β-catenin pathway of osteoblast.

It could be speculated that the mechanism of COL-HA in osteoblast may not be the Wnt/β-catenin pathway, but RUNX2 could be stimulated in BMP2 pathway by COL-HA to regulate osteoblast differentiation. The regulation of COL-HA in osteoblast was shown in Figure 7B.

## 4 Conclusion

In this work, collagen-hyaluronate composite gradient membrane was prepared via unidirectional freeze-drying technique and sodium hyaluronate cross-linking technique and exhibited excellent mechanical strength, hydrophilicity and biocompatibility, which could facilitate cell adhesion and differentiation. Remarkably, COL-HA could effectively activate TGF-β/Smad signaling pathway in fibroblast to guide tissue regeneration, and activate BMP2 signaling pathway in osteoblast to regulate osteoblast differentiation. Thus, COL-HA could be an effective candidate for GTR application.

## Data Availability

The raw data supporting the conclusion of this article will be made available by the authors, without undue reservation.
